# Evaluation of conditional treatment effects of adjuvant treatments on patients with synovial sarcoma using Bayesian subgroup analysis

**DOI:** 10.1186/s12911-020-01305-9

**Published:** 2020-12-03

**Authors:** Sung Wook Seo, Jisoo Kim, Jihye Son, Sungbin Lim

**Affiliations:** 1 Department of Orthopedic Surgery, Samsung Medical Center, Sungkyunkwan University School of Medicine, Seoul, Korea; 2 Department of Industrial Engineering, Artificial Intelligence Graduate School, UNIST, Ulsan, Korea

**Keywords:** Synovial sarcoma, Bayesian subgroup analysis, Radiation therapy, Chemotherapy

## Abstract

**Background:**

The impact of adjuvant chemotherapy or radiation therapy on the survival of patients with synovial sarcoma (SS), which is a rare soft-tissue sarcoma, remains controversial. Bayesian statistical approaches and propensity score matching can be employed to infer treatment effects using observational data. Thus, this study aimed to identify the individual treatment effects of adjuvant therapies on the overall survival of SS patients and recognize subgroups of patients who can benefit from specific treatments using Bayesian subgroup analyses.

**Methods:**

We analyzed data from patients with SS obtained from the surveillance, epidemiology, and end results (SEER) public database. These data were collected between 1984 and 2014. The treatment effects of chemotherapy and radiation therapy on overall survival were evaluated using propensity score matching. Subgroups that could benefit from radiation therapy or chemotherapy were identified using Bayesian subgroup analyses.

**Results:**

Based on a stratified Kaplan–Meier curve, chemotherapy exhibited a positive average causal effect on survival in patients with SS, whereas radiation therapy did not. The optimal subgroup for chemotherapy includes the following covariates: older than 20 years, male, large tumor (longest diameter > 5 cm), advanced stage (SEER 3), extremity location, and spindle cell type. The optimal subgroup for radiation therapy includes the following covariates: older than 20 years, male, large tumor (longest diameter > 5 cm), early stage (SEER 1), extremity location, and biphasic type.

**Conclusion:**

In this study, we identified high-risk patients whose variables include age (age > 20 years), gender, tumor size, tumor location, and poor prognosis without adjuvant treatment. Radiation therapy should be considered in the early stages for high-risk patients with biphasic types. Conversely, chemotherapy should be considered for late-stage high-risk SS patients with spindle cell types.

## Background

Synovial sarcoma (SS) is a rare soft-tissue sarcoma that accounts for 6% of all soft-tissue sarcomas [1–3]. Its clinical presentations, including tumor size, location, and histological subtype, are diverse and significantly affect prognoses [4]. Because SS is considered as a high-grade sarcoma with poor prognosis, the role of multimodal treatment in patients with SS is heavily debated [1–4].

The effects of adjuvant chemotherapy or radiation therapy (RT) on survival in patients with SS remain controversial because definitive evidence from randomized trials is unavailable [3, 5, 6]. Therefore, treatment benefits for rare types of cancer are challenging to identify based on definitive evidence, because extensive randomized clinical trials (RCTs) are difficult to conduct [5] and subgroup analyses are often inadequately representative due to their small sample sizes.

Significant advances in statistics and data science have allowed us to address these issues. First, average treatment effects can be inferred from observational data using propensity score matching (PSM). PSM [7, 8] allows us to compare patients with similar distributions of baseline covariates, thereby minimizing the effects of confounders.

Second, statistical approaches have yielded reliable results in studies with small sample sizes [9–11]. Bayesian statistics infer the posterior distributions of treatment outcomes based on current observation results and prior beliefs. Thus, using Bayesian statistical approaches for subgroup analysis can help realize practical and credible results.

We aimed to evaluate the average treatment effects of RT and chemotherapy in SS patients, using the PSM method. Additionally, we attempted to identify specific subgroups of patients who could benefit from RT or chemotherapy, using Bayesian subgroup analyses.

We obtained the data of SS patients from the surveillance, epidemiology, and end results (SEER) database. Subsequently, we (a) evaluated differences between the survival outcomes of treated and untreated covariate-balanced patients and (b) identified subgroups that could benefit from RT or chemotherapy.

## Methods

### Study population

In the SEER database, we identified all patients with a pathologically confirmed diagnosis of SS (ICD-O-3 codes 9040, 9041, 9042, and 9043) between 1984 and 2014.

For the analysis, we collected data regarding age at diagnosis, sex, primary tumor site (axial or extremity), tumor size (large or small with a cutoff of 5 cm, according to the protocol of the American Joint Committee on Cancer), histologic subtype [spindle cell type, biphasic type, or not otherwise specified (NOS)], SEER stage (localized, regional, or distant), surgical treatment, RT, and chemotherapy as baseline covariates. The overall survival time in months and event (death or alive) data were also collected. We excluded patients with missing treatment information. Our cohort selection was conducted as follows. A total of 2249 patients were identified from 1984 to 2014. Among these patients, 712 with missing treatment information were excluded. Therefore, 1537 patients were included for the analysis (Table 1.). The missing variables were imputed using K-nearest neighbors imputation.

**Table 1:** Baseline covariates of the dataset

	Total cases (1537)	Radiation treatment				Chemotherapy treatment			
		Unmatched		Matched		Unmatched		Matched	
		RT (913)	No RT (624)	Factual RT (560)	Counter factual RT (560)	Chem (736)	No Chem (801)	Factual Chem (720)	Counter factual Chem (720)
Age	38.23 (± 18.27)	37.35 (± 17.55)	39.51 (± 19.22)	39.89 (± 18.54)	38.57 (± 17.82)	35.08 (± 15.52)	41.11 (± 20.06)	35.25 (± 15.41)	34.05 (± 13.92)
Sex (female, %)	51.20	50.49	52.24	49.46	52.14	52.31	50.19	52.78	50.56
Size (%)	61.48	62.87	59.46	58.04	58.75	76.90	47.32	76.67	79.31
Primary site (%)	76.84	77.22	76.28	76.43	77.50	74.86	78.65	74.86	72.78
SEER (%)									
Blank	1.82	1.31	2.41	0.00	0.00	1.09	3.31	0.00	0.00
1	61.74	67.36	53.61	50.00	57.32	52.85	65.01	54.03	46.53
2	23.49	22.89	24.40	26.07	26.25	25.54	21.76	25.97	27.08
3	12.95	08.43	19.58	23.93	16.43	20.52	09.92	20.00	26.39
Surgery (%)	89.92	93.54	84.62	82.14	88.21	86.14	93.38	86.11	82.78
ICD_NOS (%)	47.89	38.55	45.35	47.68	43.75	39.54	42.95	39.72	36.81
ICD_Spindle (%)	41.31	36.47	32.53	29.11	33.04	36.82	33.08	36.67	41.53
ICD_bi (%)	34.87	25.08	22.44	23.21	23.57	23.64	24.34	23.61	21.81

For external validation, we compared the survival outcomes between treated and untreated patients in subgroups of Korean SS patients. Between March of 2001 and February of 2013, data from 242 SS patients were collected from three different institutes: Seoul National University (107 patients), Samsung Medical Center (83 patients), and the National Cancer Center (52 patients). Data usage was approved by the institutional review boards of the involved institutions [Seoul National University Hospital (H-1701-084-823), Samsung Medical Center (No. 201701136), and the National Cancer Center (No. 201700190001)] (Table 2).

**Table 2:** Population of the external dataset

Patient population		242
Age	Mean	37.6 ± 2.1
Sex (%)	Male	47.93
	Female	52.07
Size (%)	Less than 5 cm	45.87
	Greater than 5 cm	54.13
Tumor location (%)	Trunk	31.82
	Extremity	68.18
SEER tumor stage (%)	1	75.62
	2	10.33
	3	14.05
Surgery (%)	No surgery	0.00
	Surgery	100.00
Radioactive treatment (%)	Untreated	38.84
	Treat	61.16
Chemical treatment (%)	Untreated	44.21
	Treat	55.79
Pathological subtype (%)	Mono	25.62
	Bi	44.63
	Unclassified	29.75

### Identification of treatment effects

In observational data, treatments are not assigned equally because variables known as confounders can affect the assignment of treatments. For example, late-stage cancer patients are more likely to be administered chemotherapy; however, these patients are also more strongly associated with poor prognoses. If the stages are unequally matched, we may derive a biased conclusion that chemotherapy is closely associated with poor prognoses. Therefore, we need to compare outcomes between treated and untreated individuals only when they have similar variables or are in the same unit ($u_{i}$, as defined by Pearl with the backdoor criterion). For this purpose, we divided the study population into units or subgroups with identical variables ($u_{i}$). Thereafter, we evaluated treatment effects based on the covariates of each unit. Such effects are referred to as conditional treatment effects (CTEs). Under the stable unit treatment value assumption [12], the CTE (X) of a unit subgroup ($u_{i}$) can be defined as follows:

$$Y \, (T = 1,u_{i} ),Y \, (T = 0,u_{i} ) \bot u_{i} ,u_{i} \in {\mathbb{S}} \left( {subgroups} \right).$$

The treatment effect of a subgroup ($u_{i}$) can be denoted as a CTE assuming that $x \cong x^{\prime}, \left( {x,x^{\prime} \in u_{i} } \right).$ The CTE τ ($x \in u_{i}$) of a subject (T $= 1, x \in u_{i}$) can be defined as follows:

$${\Gamma }_{CTE} (.|u_{i} ) = {\mathbb{E}}[Y \, (T = 1|x) - Y \, (T = 0|x^{^{\prime}} )|x, x^{^{\prime}} \in u_{i} ].$$

Therefore, treatment outcomes can be compared among the subjects within a unit. Here, we randomly selected subjects from each unit and compared their outcomes using win probabilities.

### Subgroup clustering

We divided the study population into units or subgroups with identical variables using a hierarchical clustering method. By considering all possible combinations of nine variables—age, sex, tumor size, location, SEER stage, surgery, RT or chemotherapy, spindle cell type, and biphasic type—we divided the patients into 512 subgroups ($2^{9}$).

### Bayesian subgroup analysis

The outcome of a treatment (Y) was defined as the win probability (i.e., chance of a treated patient to live longer than untreated patients). The survival times of treated and untreated patients in each subgroup ($u_{i}$) were compared using the concordance method, which is a ranking method for identifying survival winners by matching each patient with the other patients in the same group. The win probability of the observed patients followed a binomial likelihood distribution P(X|Y), and the beta distribution was the conjugate prior to the binomial likelihood distribution. Prior knowledge P(Y) was defined as a beta distribution Beta (α,β). As we did not possess any prior knowledge regarding the treatment outcomes of each subgroup, we considered a uniform prior (α = 1, β = 1) for the prior P(Y). The expected posterior probability of treatment outcomes in a subgroup ($u_{i}$) can be updated by observing data, as follows:

$$\begin{aligned} P\left( {Y|X} \right) P(X|Y) P\left( { Y} \right) \\ & Prior \,P\left( Y \right) \sim Beta\, \left( , \right) \\ & Posterior\, P\left( {Y|X} \right) \sim Beta\, \left( { + , + {\text{n}} - } \right) \\ & {\text{P}}({\text{X}}|{\text{Y}}):likelihood\,\, or \,\,win \,\,probability \\ & {\text{X }} \in u_{i} :{\text{ a }}\,\,patient\,\, in \,\,the\,\, subgroup\,\, with\,\, the\,\, same \,\,covariates \\ & Y \in {\mathbb{R}},\,\,0 \le Y \le 1 : \,\,treatment\,\,benefit \\ & \gamma :number \,\,of\,\, win{\text{s}} \\ & n:number \,\,of\,\,observations \\ & Beta\, \left( , \right):\,\,{\text{prior }}\,\,{\text{beta }}\,\,{\text{distribution }} \\ & {\text{Y}}:\,\,{\text{treatment}}\,\,{\text{outcome}}. \\ \end{aligned}$$

Subgroups for which the Bayes factor (BF) was higher than three (substantial evidence according to Kass and Raftery [13]) were selected as the credible subgroups. The most credible subgroup for the treatment was defined as the group for which the lower bound of the 95% credibility interval [95% confidence interval (CI) of treatment benefit] was the highest.

The net treatment benefits of optimal subgroups $({\mathbb{S}}$*) can be estimated by comparing the CTEs of optimal groups and other subgroups (${\mathbb{S}} \ne {\mathbb{S}}$*), as follows:

$$\begin{aligned} & Treatment \, Benefit = {\text{CTE}}_{{{\mathbb{S}}{*}}} - {\text{CTE}}_{{\left( {{\mathbb{S}} \ne {\mathbb{S}}{*}} \right) }} \\ & \quad = {\mathbb{E}}[Y \, (T = 1|x) {-}Y \, (T = 0|x^{\prime})|x, x^{^{\prime}} \in {\mathbb{S}}^{*} ] \\ & \quad - {\mathbb{E}}[Y \, (T = 1|x) - Y(T = 0|x^{\prime})|x, x^{^{\prime}} \notin {\mathbb{S}}^{*} ]. \\ \end{aligned}$$

Pairs of distributions (${\text{CTE}}_{{{\mathbb{S}}{*}}}$ vs. ${\text{CTE}}_{{\left( {{\mathbb{S}} \ne {\mathbb{S}}{*}} \right)}}$) were compared by plotting 2D bivariate distributions. If approximately 95% of the area of a bivariate distribution is located on the upper-left side of the neutral line, then this indicates that a treatment has a more significant benefit for the optimal subgroups compared to that for other subgroups.

### Statistical methods

PSM is used to match sets of patients who share the same propensity score (27 units of score with a standard deviation of 0.2). Because a propensity score represents a probability of treatment assignment, we can assume that patients in a matched set are independent of treatment conditions [14]. We adopted a logistic regression method whereby treatment assignments were regressed based on the nine baseline variables. Survival outcomes were evaluated using Kaplan–Meier survival analyses. A stratified log-rank test was used to compare the survival curves of matched patients. Statistical analysis was performed using the Statistical Package for the Social Sciences software version 23 (IBM Corp., La Jolla, CA, US). Bayesian statistical modeling was conducted using a Python package called PyMC3. The Scikit-learn library was used for preprocessing data. The results are presented as mean ± 95% CI, unless otherwise specified.

## Results

### Average treatment effects on the survival of SS patients

#### Impact of chemotherapy on the survival of SS patients

**Fig. 1:**
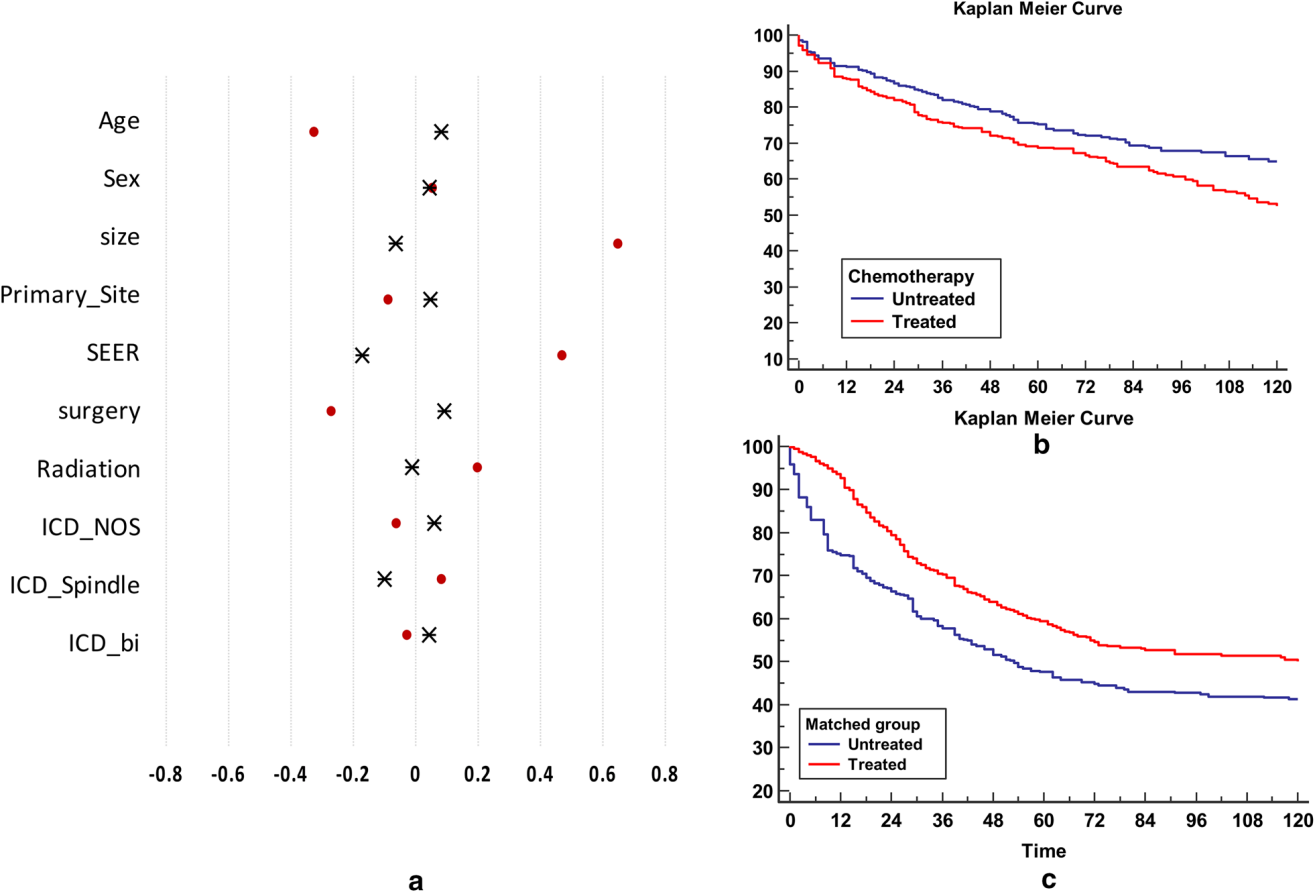
Overall treatment effects of chemotherapy. **a** Standard difference in baseline covariates between treated and untreated patients. The covariates of unmatched patients (red dot) represent significant differences between the treated and untreated groups. The treated patients are more likely to be younger and have a larger tumor size and higher SEER stage compared to the untreated patients. Following propensity matching, the covariates are well balanced (black star). **b** Kaplan–Meier survival curves representing the overall survival rate of patients with SS. The treated group exhibits worse prognoses compared to the untreated group [*p* = 0.003, hazard ratio: 1.32 (95% CI: 1.10–1.59)]. The x-axis represents time in months. **c** Kaplan–Meier survival curves of matched patients with SS. The treated group exhibits significantly better prognoses than the untreated group (*p* = 0.0001). The x-axis represents time in months

Data from all treated and untreated patients were used to approximate the propensity function of chemotherapy. The baseline covariates in the dataset are listed in Table 1. Systemic differences in terms of baseline variables can be observed between the treated and untreated patients, as shown in Fig. 1a. Each of the 736 patients who underwent chemotherapy were matched with the 801 untreated patients with propensity scores similar to those of chemotherapy, in order to estimate the average treatment effect for the entire population. The results indicate that chemotherapy is significantly beneficial for survival in all patients (*p* = 0.0001; Fig. 1b, c).

#### Impact of RT on the survival of SS patients

**Fig. 2:**
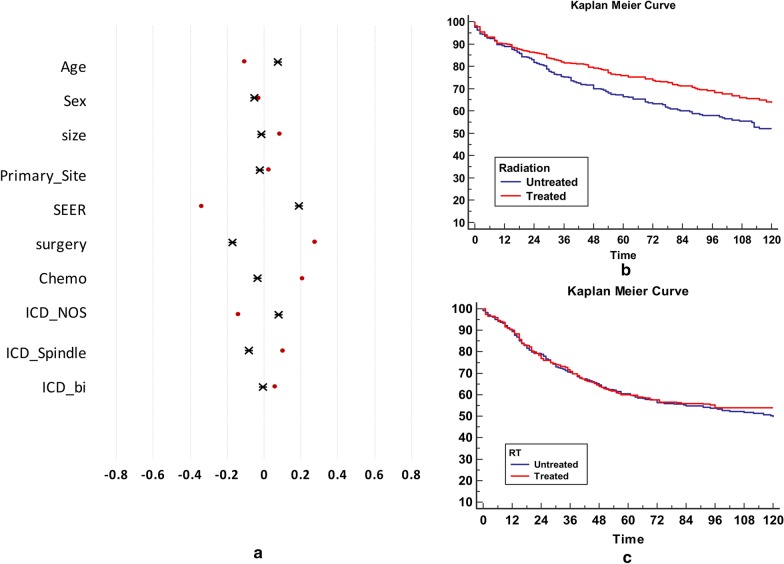
Overall treatment effects of RT. **a** Standard difference in baseline covariates between the treated and untreated patients. The covariates of unmatched patients (red dot) represent significant differences between the treated and untreated groups. The treated patients are more likely to have a lower SEER stage and be surgically treated compared to the untreated patients. Following propensity matching, the covariates are well balanced (black star). **b** Kaplan–Meier survival curves for the overall survival of patients with SS. The treated group exhibits significantly better prognoses compared to the untreated group (*p* = 0.0014). The x-axis represents time in months. **c** Kaplan–Meier survival curves of matched patients with SS. The treated group exhibits similar prognoses compared to the untreated group (*p* = 0.56). The x-axis represents time in months

Data from all treated and untreated patients were used to approximate the propensity function of RT. Each of the 913 patients who were treated with RT were matched with the 624 untreated patients with propensity scores similar to those of RT, to estimate the average treatment effect for the entire population. The baseline characteristics and standardized differences of the matched samples for RT are presented in Table 1 and Fig. 2a. These results indicate that RT is not significantly beneficial for survival in all patients (Fig. 2b, c).

### CTEs on the survival of SS patients in subgroups

Patients were divided into 512 subgroups using the hierarchical clustering method discussed earlier. The win probability [$${\Gamma }_{CTE} \left( {X| u_{i} \in {\mathbb{S}}} \right) ]$$ of each patient was updated in the beta prior distribution of each subgroup ($$u_{i} )$$. Subsequently, we identified the optimal subgroup $$({\mathbb{S}}^*)$$ for which BF was greater than three and the lower bound (95% CI) of the win probability distribution for the treatment group was greater than 0.5 (50% win probability).

#### Impact of chemotherapy on the survival of SS patients in subgroups

**Fig. 3:**
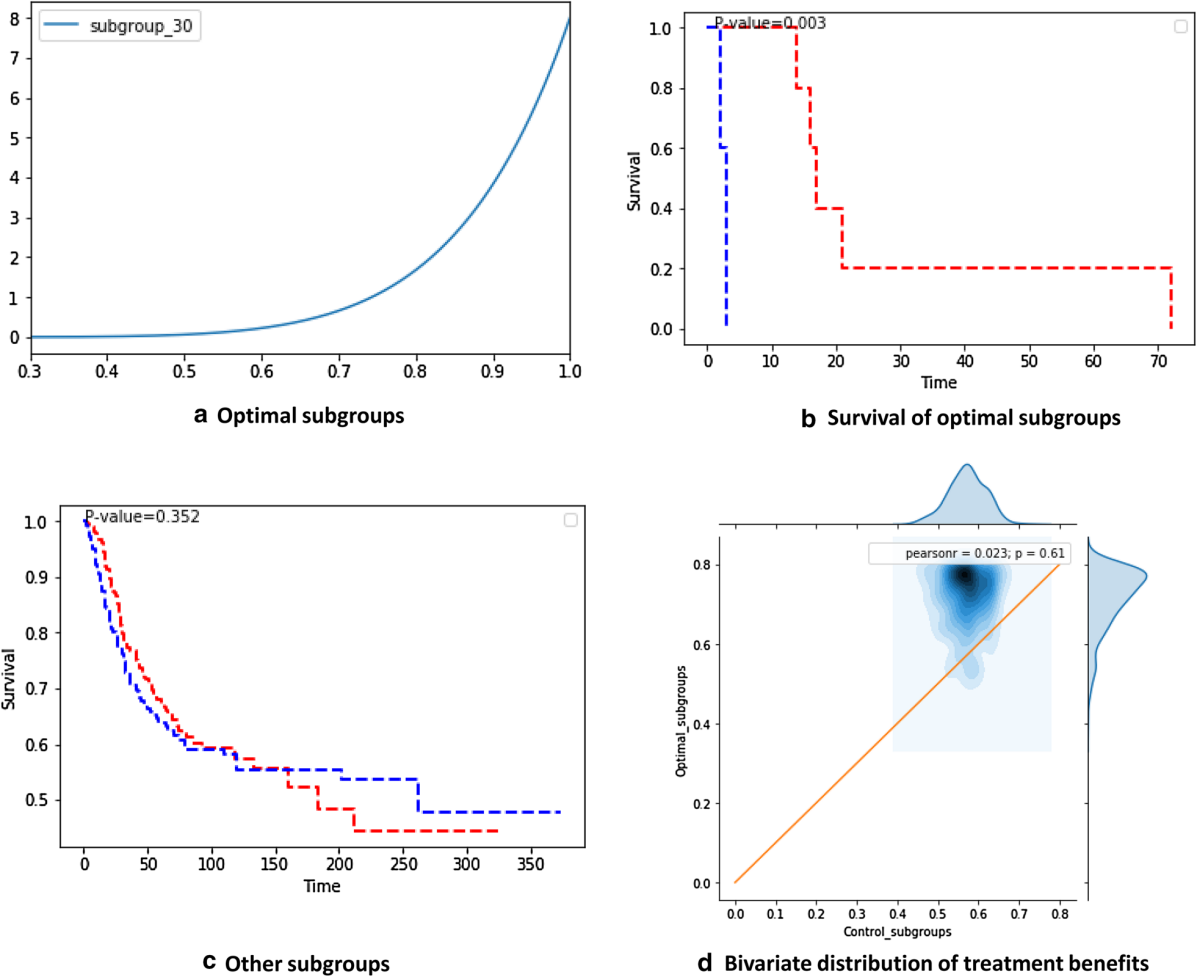
Bayesian inference of the CTEs of chemotherapy for the optimal subgroup. **a** Win probability distribution of the optimal subgroup. In subgroup 30, the patients who were treated with chemotherapy exhibit a significantly greater probability of survival compared to the untreated patients (lower bound of 95% CI > 0.5). **b** Kaplan–Meier survival curves of optimal subgroups. The treated patients exhibit significantly better prognoses than the untreated patients in the subgroups (*p* = 0.003). **c** Kaplan–Meier survival curves of other subgroups outside the optimal subgroup. The treated patients do not exhibit significantly better prognoses than the untreated patients in these subgroups (*p* = 0.352). **d** Bivariate win probability distribution for visualizing treatment benefits. To compare the distributions of win probabilities between the optimal and control subgroups, we visualized the joint distribution of these two groups. The blue contour represents the density of probability and the end of the contour (margin of the graph) represents a 95% CI. The upper triangle represents the area of treatment benefit

**Fig. 4:**
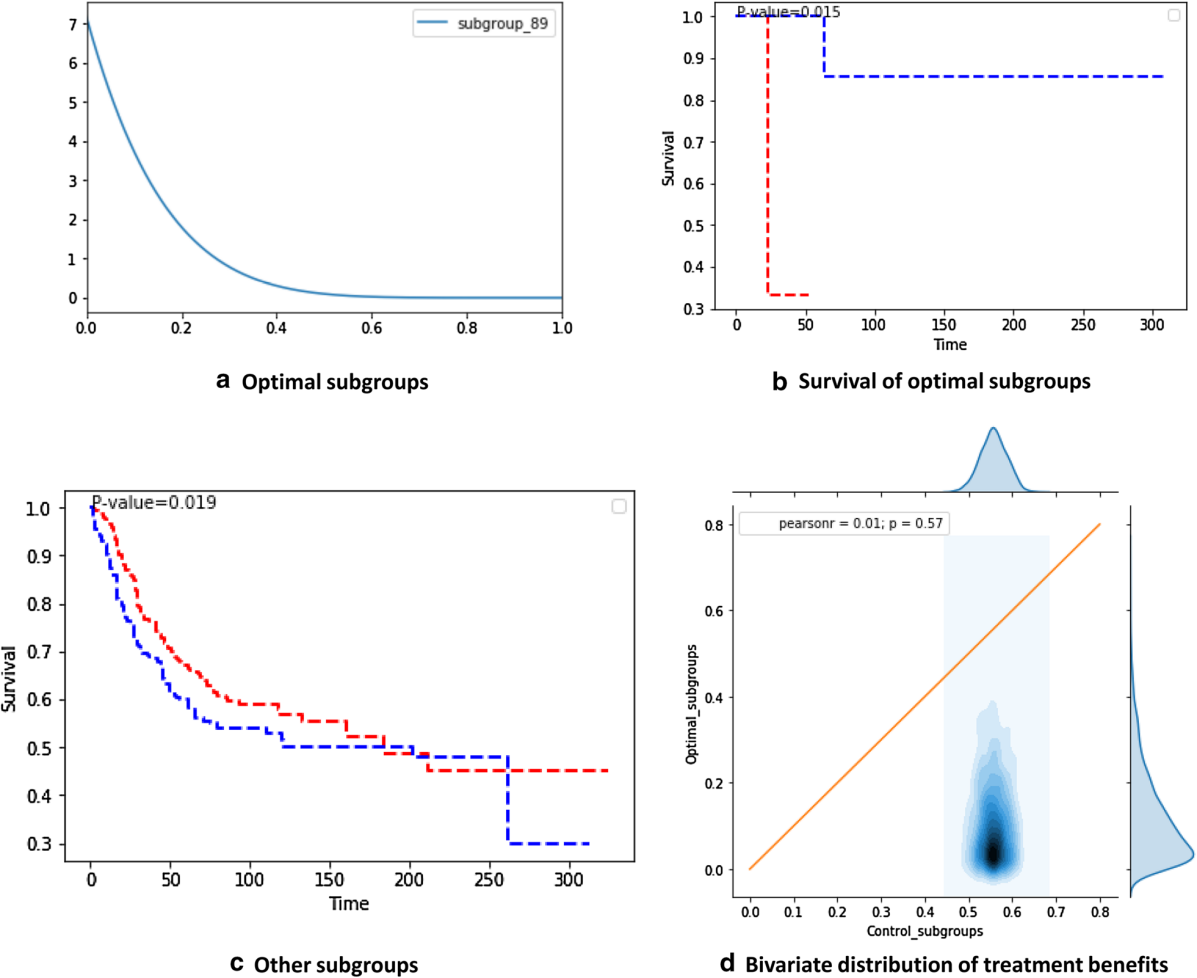
Bayesian inference of the CTEs of chemotherapy for the worst subgroup. **a** Win probability distribution of the worst subgroup. In subgroup 89, patients who were treated with chemotherapy exhibit a significantly greater probability of survival compared to the untreated patients (lower bound of 95% CI > 0.5). **b** Kaplan–Meier survival curves of the worst subgroup. The treated patients exhibit significantly better prognoses than the untreated patients in this subgroup (*p* = 0.015). **c** Kaplan–Meier survival curves of other subgroups outside the worst subgroup. The treated patients do not exhibit significantly better prognoses than the untreated patients in these subgroups (*p* = 0.019). **d** Bivariate win probability distribution for visualizing treatment benefits. To compare the distributions of win probabilities between the optimal and control subgroups, we visualize the joint distribution of these two groups. The blue contour represents the density of probability and the end of the contour (margin of the graph) represents a 95% CI. The upper triangle represents the area of treatment benefit

One subgroup was identified as the optimal subgroup for chemotherapy, where BF was greater than three and the win probability (CTE) of chemotherapy was greater 50% (95% CI) (Fig. 3a). This subgroup includes male patients who are older than 20 years and have large tumors (longest diameter > 5 cm) in their extremities, an advanced stage of cancer (SEER 3), and spindle cell type of SS. Based on the corresponding Kaplan–Meier curves, the treated patients exhibited significantly better prognoses than the untreated patients in the optimal subgroup ${\mathbb{S}}$* (*p* = 0.003) (Fig. 3b). In this subgroup, the untreated patients have a 5.8 times greater risk of death compared to the treated patients [hazard ratio: 5.8 (95% CI: 0.29–17.8)]. However, the Kaplan–Meier curves indicated an insignificant difference between the prognoses of treated and untreated patients in the other subgroups (*p* = 0.352). The two distributions (${\text{CTE}}_{{{\mathbb{S}}{*}}}$ vs. ${\text{CTE}}_{{\left( {{\mathbb{S}} \ne {\mathbb{S}}{*}} \right)}}$) were compared by plotting a 2D bivariate distribution. Approximately 95% of the area of the bivariate distribution was located on the upper-left side of the neutral line. Therefore, the results indicate that chemotherapy significantly enhanced the benefit for this subgroup, as compared to the other subgroups (Fig. 3d). The worst subgroup for chemotherapy was also isolated, where BF was greater than three and 95% of the win probability distribution for chemotherapy was below 50% (Fig. 4a). This subgroup includes male patients who are older than 20 years; underwent RT; and have large tumors (longest diameter > 5 cm) located in their trunks, an early stage of cancer (SEER 1), and spindle cell type of SS. In this subgroup, chemotherapy patients exhibited significantly poorer prognoses than untreated patients (*p* = 0.015) (Fig. 4b). However, chemotherapy generally improved prognoses in the other subgroups (*p* = 0.019) (Fig. 4c). Approximately 95% of the area of the corresponding bivariate distribution was located on the lower-right side of the neutral line (Fig. 4d). Therefore, these results indicate that treatment without chemotherapy has a significantly enhanced benefit for this subgroup, as compared to the other subgroups. For this subgroup, the risk of death increased by nine times when patients underwent chemotherapy [hazard ratio: 9.0 (95% CI: 0.48–169.2)].

#### Impact of RT on the survival of SS patients in subgroups

**Fig. 5:**
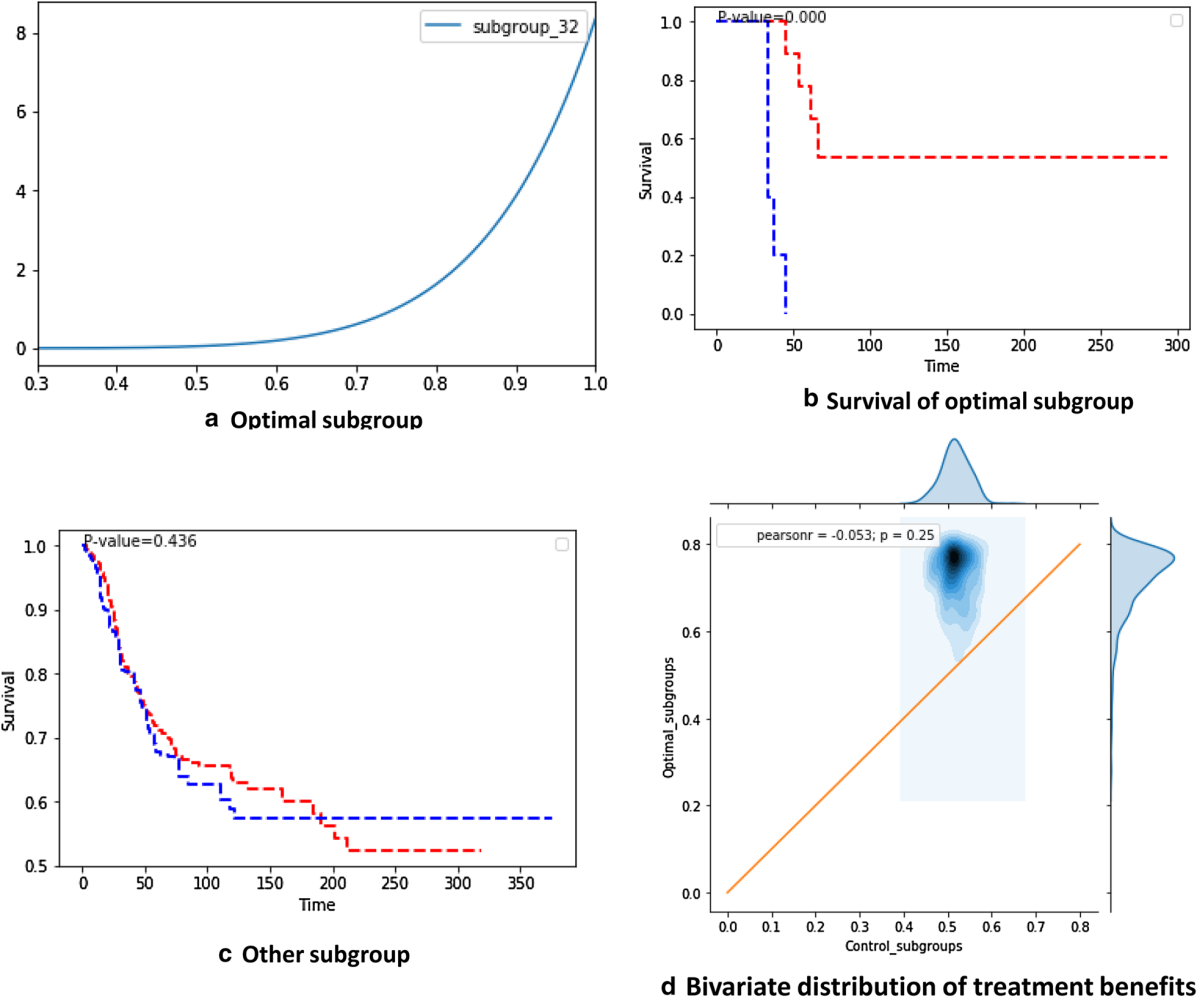
Bayesian inference of the CTEs of RT. **a** Win probability distribution of the optimal subgroup. In subgroup 32, the patients who were treated with chemotherapy exhibit a significantly greater probability of survival compared to the untreated patients (lower bound of 95% CI > 0.5). **b** Kaplan–Meier survival curves of the worst subgroup. The treated patients exhibit significantly better prognoses than the untreated patients in this subgroup (*p* = 0.000). **c** Kaplan–Meier survival curves of other subgroups outside the worst subgroup. The treated patients do not exhibit significantly better prognoses than the untreated patients in these subgroups (*p* = 0.436). **d** Bivariate win probability distribution for visualizing treatment benefits. To compare the distributions of win probabilities between the optimal and control subgroups, we visualize the joint distribution of these two groups. The blue contour represents the density of probability and the end of the contour (margin of the graph) represents a 95% CI. The upper triangle represents the area of treatment benefit

One subgroup was identified as the optimal subgroup for RT, where BF was greater than three and the win probability (CTE) of RT was greater than 50% (95% CI) (Fig. 5a). This subgroup included male patients who were older than 20 y and have large tumors (longest diameter > 5 cm), early stages of cancer (SEER 1) in their extremities, and a biphasic type of SS. The Kaplan–Meier curves indicate that treated patients have significantly better prognoses compared to untreated patients in the optimal subgroup ${\mathbb{S}}$* (*p* = 0.000) (Fig. 5b). In this subgroup, untreated patients have a 5.1 times greater risk of death compared to treated patients [hazard ratio: 5.1 (95% CI 0.31–85.3)]. However, the Kaplan–Meier curves do not indicate any significant differences between the prognoses of treated and untreated patients in the other subgroups (*p* = 0.0802).

To estimate the net treatment benefits for the optimal subgroups ${\mathbb{S}}$*, we compared the two probability distributions (${\text{CTE}}_{{{\mathbb{S}}{*}}}$ vs. ${\text{CTE}}_{{\left( {{\mathbb{S}} \ne {\mathbb{S}}{*}} \right)}}$) using a 2D bivariate distribution. We found that 95% of the area of the bivariate distribution was located on the upper-left side of the neutral line. This indicates that treatment has a more significantly enhanced benefit for the optimal subgroup, as compared to that for the other subgroups (Fig. 5d). The worst subgroup for RT satisfying the required evidence level (BF greater than three and 95% of the win probability distribution below 50%) could not be identified from the available data.

#### External validation

**Fig. 6:**
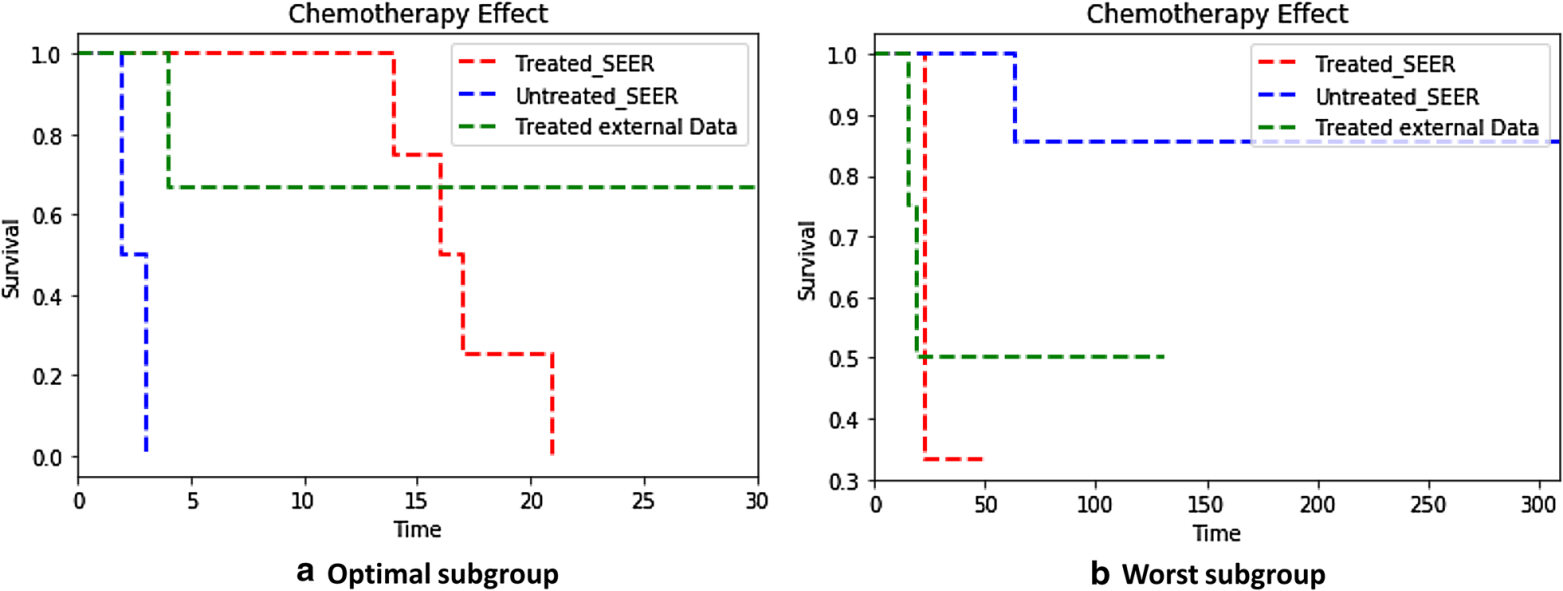
Survival outcomes in the subgroups of the external data. **a** Kaplan–Meier survival curves of the optimal subgroup for chemotherapy. The treated patients in the validation set and untreated patients in the SEER data exhibit significant differences in terms of survival rates (*p* = 0.039). **b** Kaplan–Meier survival curves of the worst subgroup for chemotherapy. The treated patients in the validation set and untreated patients in the SEER data exhibit no significant differences in terms of survival rates (*p* = 0.128). However, the treated patients exhibit a similarly poor prognosis compared to the treated patients in the same subgroup in the SEER dataset

Based on SEER data, we identified the optimal subgroup for chemotherapy, worst subgroup for chemotherapy, and optimal subgroup for RT. For external validation, we isolated the same subgroups from an external data set (Table 2) and compared the outcomes between the treated and untreated patients in each subgroup using Kaplan–Meier survival analyses. All the patients in the optimal subgroup for chemotherapy in the validation set underwent chemotherapy. Therefore, we compared the treated patients from this dataset with the patients in the SEER dataset. The treated patients in the validation set did not exhibit any significant differences in terms of survival time compared to the treated patients in the SEER dataset (*p* = 0.221), but did exhibit significant differences in terms of survival time compared to the untreated patients in the SEER dataset (*p* = 0.039) (Fig. 6a).

Moreover, all the patients in the worst subgroup for chemotherapy in the validation set underwent chemotherapy. Therefore, we compared these treated patients to the patients in the SEER dataset. The treated patients in the validation set did not exhibit any significant differences in terms of survival time compared to the treated (*p* = 0.889) and untreated (*p* = 0.128) patients in the SEER dataset. Although the differences were not statistically significant due to the small sample size of the validation set, the survival curve of the validation set indicated poorer prognoses compared to the untreated patients in this subgroup (Fig. 6b). The optimal subgroup for RT in the validation set contained only one patient who underwent RT. Therefore, we could not compare the outcomes for this group.

## Discussion

The effects of adjuvant treatment on patients with SS are difficult to evaluate because the incidence of this type of sarcoma is considerably low. Therefore, the use of adjuvant treatments, such as chemotherapy or RT, for patients with SS remains controversial. Based on our results, we conclude that, for the unbalanced baseline covariates, chemotherapy appears to be associated with poor prognoses. However, based on the balanced data, we determined that chemotherapy has a positive effect on survival rates. Propensity matching allows us to balance covariates between the two groups at the complete sample level, which enables us to estimate average treatment effect [15].

Average causal effects do not necessarily indicate that the same treatment effects can be expected for all patients. In fact, treatment effects may vary for a substantial subgroup. Bayesian subgroup analysis is a reasonable method for determining the optimal subgroups for a given treatment, because we can compare treatment effects within subgroups, wherein the covariates of patients are similar [16]. Therefore, we can estimate the CTEs and credibility (or credible intervals) for each subgroup, which are crucial for clinical decision making. We define a CTE as a binary outcome (effect = 1 and no effect = 0) and measure the posterior beta distribution of each CTE, where the win probability is updated based on the individuals in each subgroup. Because the number of cases in each subgroup determines the corresponding credible intervals, we can quantify the evidence level and uncertainty of treatment effects for each subgroup. Therefore, we can identify an optimal subgroup with credible evidence by considering ranges of uncertainty. For other subgroups, SEER data is insufficient and additional evidence must be obtained.

Bayesian subgroup analysis is useful for identifying an optimal experimental subgroup for a clinical trial, which is called an adaptive clinical trial, instead of simply enrolling all patients. This method does not enroll a subgroup of patients for which the evidence of treatment effects is clear based on past data; instead, it selects only a subset of patients for which the evidence is still weak [17]. Therefore, we can reduce the number of participants required for RCTs and improve the likelihood of detecting treatment effects, which can help overcome the ethical issues of RCTs.

We should note some limitations of this study. Although the SEER data that we used in this study may represent the largest dataset of SS patients, the number of variables is still limited and some of the treatment information is unclear. Furthermore, some unmeasured confounding variables that were not corrected by PSM may be present.

The large sample size used in our study may overcome the limitations of selection bias and a lack of generalizability, which are potential weaknesses of single-institution studies.

The probability of incorrect specifications of the subgroups is low, because SEER data provide large sample sizes, which enables more precise subgroup clustering considering all the possible risk factors. SEER data also overcome any lack of generalizability, which is a potential weakness in single-institution studies.

Another limitation is that the sample size of our external data is smaller than that of SEER data, and there is lack of samples for a few subgroups. In the external observation data, the high-risk group tends to be treated using chemotherapy, and there are no untreated cases for comparison. Therefore, we could not directly compare the outcomes of treated patients to those of untreated patients within a subgroup using the external dataset. However, the outcomes of the SEER data and external data were not significantly different under similar conditions, such as similar subgroups and treatments.

In this study, we determined that chemotherapy is effective for an optimal subgroup with characteristics of ages greater than 20 years, male, large tumors (longest diameter > 5 cm), extremity locations, SEER stage 3, spindle cell type, and treated with surgery, without RT. Previous studies have found that sex (male) [18] and a non-biphasic subtype [18, 19] are strongly associated with poor prognoses. However, the association between these factors and the treatment effects has not been evaluated. Although we could not identify specific regimes of chemotherapy from the SEER data, our results highlight the importance of systemic chemotherapy in such poor prognostic subgroups.

In a study on national practice patterns for soft-tissue sarcoma, it was determined that SS patients have a relatively high likelihood of receiving chemotherapy [20]. This study highlighted the fact that multimodal therapy, including chemotherapy, may increase the severe toxicity in adults and limit any incremental benefits in terms of long-term outcomes. Therefore, an appropriate selection of patients for chemotherapy is crucial. Our Bayesian subgroup analysis isolated the worst subgroup for which chemotherapy significantly reduced the survival time compared to untreated patients. This subgroup exhibits prognostic factors such as old age (> 20 years), male, large tumors (> 5 cm), and spindle cell type, which are the same as those of the optimal subgroup. However, this subgroup also includes the early stages of cancer (SEER stage 1), tumors located in the trunk or pelvis, and patients treated with both surgery and RT. Our study provides the first evidence that chemotherapy may not be suitable for all patients with poor prognostic factors. If the SS is located in the trunk or pelvis and in the early stages, chemotherapy may increase mortality or morbidity. Therefore, surgery combined with RT should be considered as an optimal treatment for this subgroup.

Other studies have shown that RT is associated with good prognoses in patients with high-grade sarcoma [21, 22]. Adjuvant RT reportedly improves five-year local-recurrence-free survival rates [23]. Other studies have also revealed improved local control and disease-free survival with RT [24, 25]. However, the effects of RT on survival gains have been controversial because RT only controls local diseases and may have limited effect on systemic metastasis. Yang et al. failed to identify significant benefits of RT in terms of the overall survival rate among extremity SS patients [26]. Canter et al. did not identify RT as a significant factor in terms of survival outcomes [27]. Based on our results, we also could not determine the average treatment effects of RT in terms of the survival of patients with SS in propensity-score-matched cases. However, there may be a subgroup that can benefit from RT in terms of survival time. Through our Bayesian subgroup analysis, we identified an optimal subgroup with a significantly enhanced CTE. Our results indicated that RT is more effective in a subgroup with characteristics of old age (age > 20 years), male, large tumors (> 5 cm), extremity locations, early stages (SEER 1), and biphasic subtypes. Although the patients in this subgroup are all in the early stages, their survival rate is significantly lower without RT.

Although underlying biological mechanisms require additional research, our study is the first to address the treatment effects and CTEs of chemotherapy and RT in subgroups of SS. Our results are expected to be useful for clinical decision making in terms of selecting optimal subgroups for chemotherapy or RT.

## Conclusions

In this study, we identified high-risk patients whose variables include old age (age > 20 years), male, large tumors (> 5 cm), and extremity locations, and who have significantly poorer prognoses without adjuvant treatment. RT should be considered in the early stages of cancer for high-risk patients with biphasic types. Contrarily, chemotherapy should be considered for late-stage high-risk SS patients with spindle cell types.

## Data Availability

The datasets analyzed during this study are available from the SEER database (https://seer.cancer.gov/data/access.html).

## Abbreviations

SS: Synovial sarcoma
RT: Radiation therapy
RCT: Randomized clinical trial
PSM: Propensity score matching
SEER: Surveillance, epidemiology, and end results
NOS: Not otherwise specified
CTE: Conditional treatment effect
CI: Confidence interval
